# Mechanical Testing of the New Cage for Tibial Tuberosity Advancement with the Cranial Implant Fixation (TTA CF) Technique—Ex Vivo Study on Sheep Model

**DOI:** 10.3390/ani12162013

**Published:** 2022-08-09

**Authors:** Yauheni Zhalniarovich, Paulina Przyborowska-Zhalniarovich, Angelika Tobolska, Marta Mieszkowska, Justyna Abako, Magdalena Morawska-Kozłowska, Marcin Mieszkowski, Dariusz Onichimowski

**Affiliations:** 1 Department of Surgery and Radiology, Faculty of Veterinary Medicine, University of Warmia and Mazury in Olsztyn, Oczapowskiego 14, 10-718 Olsztyn, Poland; 2 Department of Anesthesiology and Intensive Care, School of Medicine, Collegium Medicum, University of Warmia and Mazury in Olsztyn, Warszawska 30, 11-082 Olsztyn, Poland

**Keywords:** cranial cruciate ligament, stifle, osteotomy, biomechanics, ovine model, dynamic stabilization

## Abstract

**Simple Summary:**

Tibial tuberosity advancement is a method of surgical treatment of cranial cruciate ligament rupture in animals.. In previous reports, the biomechanical effectiveness of tibial tuberosity advancement surgeries was evaluated by axial pressure on the tibial tuberosity to test the strength and resistance of the fixation or by pulling on the tuberosity. To our knowledge, there are no reports that examined the strength that is needed to pull out an implant from the tibia after tibial advancement. This study is the first report that focuses on pulling out the TTA implant, which corresponds to the biointegrity and ingrowth of the TTA cage with the tibia.

**Abstract:**

*Background*: Modifications of tibial tuberosity advancement are well accepted for cranial cruciate rupture repair. We compared the loads that were needed to pull the TTA CF cage out in the two groups. The first group consisted of five sheep in which osteotomy and TTA CF cage fixation were performed as assumed preoperatively. The second group consisted of five sheep in which intraoperative or postoperative discrepancies from preoperative planning were found. This is also the first report describing biomechanical testing after tibial tuberosity advancement with cranial implant fixation (TTA CF) surgical procedures. *Results*: A total of 10 ovine proximal tibiae were tested biomechanically by tearing out TTA CF implants from the bone. The mean maximal loaded forces to pull out the cage in Group 1, in which fixation of the implant was performed as assumed preoperatively, was 878 ± 61 N, and in Group 2, in which discrepancies from preoperative planning were found, was 330 ± 55 N. The mean implant displacement under maximal load to failure was 2.6 mm and 2.2 mm in Groups 1 and 2, respectively. There was a significant difference between Group 1 and Group 2 in the maximal loads-to-failure; however, the difference in the displacement at maximal loaded forces to pull out the cage was not significant between the groups. *Conclusions*: The mean maximal loaded forces to pull out the cage was significantly lower in Group 2, where discrepancies from preoperative planning were found (878 ± 61 N vs. 330 ± 55 N). The lower forces that were needed to extract the TTA CF implant from the tibia can lead to the conclusion that biointegration of the implant is also weaker. Correct positioning of the osteotomy line and TTA CF implant is essential for good biointegrity and thus for limiting complications in the form of tibial tuberosity avulsion fracture or tibial shaft fracture.

## 1. Introduction

Cranial cruciate ligament (CCL) rupture causes both rotational and translational instability of the stifle joint. Many surgical procedures have been developed to prevent tibiofemoral drawer forces using either dynamic stabilization or static stabilization techniques in dogs [1,2,3]. Tibial tuberosity advancement (TTA) and tibial plateau levelling osteotomy (TPLO), and their subsequent modifications are the most frequently used techniques for the dynamic stabilization of the stifle joint in dogs [4,5,6,7,8]. One of the recently described modifications of the TTA procedure was named TTA with cranial implant fixation (TTA CF) [9,10]. Conventional TTA and TTA CF are based on the theory that the angle between the patella tendon and the tibia plateau affects the formation of tibiofemoral shear forces during weight bearing [11]. The mechanical principle of all TTA techniques relies on the elimination of the cranial tibial thrust by the placement of the patellar ligament perpendicular to the tibial plateau [5,6]. Performing proximal mediolateral osteotomy and advancing the tuberosity of the tibia cranially provides dynamic stability to the stifle joint [5,6]. Clinical studies have proven the biomechanical principle and normal limb function after tibial tuberosity advancement surgeries [8,10,12]. 

The TTA Rapid was first reported in 2014 [8] and based on the Maquet procedure for treating patellofemoral osteoarthritis or chondromalacia of the patella [13]. TTA-2 [14] and TTA CF [9,10] are simplified modifications of TTA surgery that consists of the Maquet osteotomy, settled with a new design cage that excludes stress risers that are created by the fork, plate, and screws, hence fork and plate fixation are unnecessary [9,10,14]. Studies have confirmed the assumptions that the cage size, cage position relative to the tibial tuberosity, and the conformation of the tuberosity have an impact on the clinical outcomes and rate of complications after TTA [15]. During the TTA CF procedure, the only implants that were needed were a hollow titanium cage with porous surfaces and two 2.7 mm titanium self-taping screws that were positioned in a craniocaudal direction from the tibial crest [9,10].

Reviewing mechanical ex vivo studies about modifications or new TTA techniques, the biomechanical effectiveness was evaluated by axial pressure on the tibial tuberosity to test the strength and resistance of the fixation [16,17,18] or by pulling on the tuberosity [7,19,20] checking what force causes detachment of the tibial tuberosity. To the best of our knowledge, there are no studies in the literature that examined the strength that is needed to pull out a TTA cage from the bone. The present study is the first report examining the pull-out forces and ingrowth of the TTA CF cage with the tibia. This is also the first report describing biomechanical testing after TTA CF surgical procedures.

**Figure 1:**
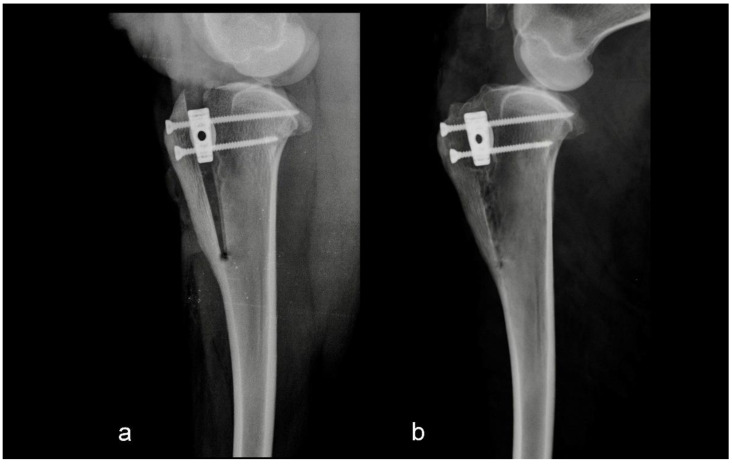
Mediolateral radiograph of the stifle in Group 1 immediately postoperatively (**a**) and at six months postoperatively (**b**). Osteotomy and tibial tuberosity advancement were performed as assumed preoperatively.

The main purpose of this retrospective biomechanical study was to determine the biointegrity of porous TTA CF cages in the tibia six months after surgery. The other purpose was to compare the loads that were needed to pull the TTA CF cage out in the two groups. The groups were divided retrospectively depending on the postoperative radiographs. The first group consisted of five sheep in which osteotomy and TTA CF cage fixation were performed as assumed preoperatively (Figure 1).

**Figure 2:**
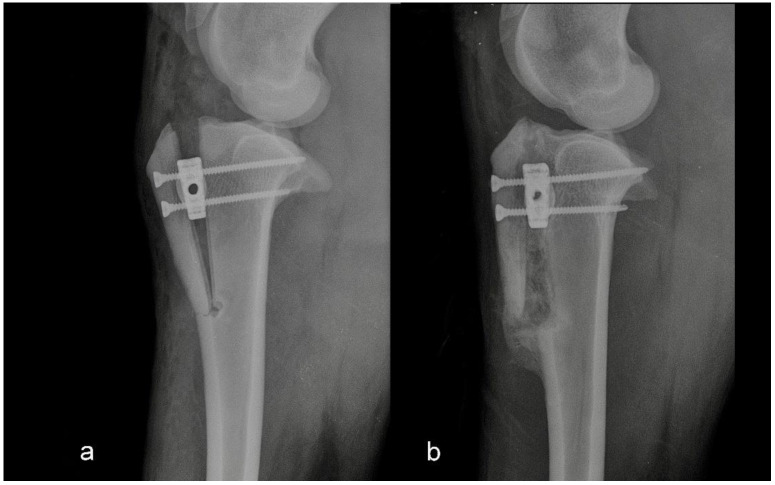
Mediolateral radiograph of the stifle in Group 2 immediately postoperatively (**a**) and at six months postoperatively (**b**). The tibial crest was cracked at the level of the Maquet hole.

**Figure 3:**
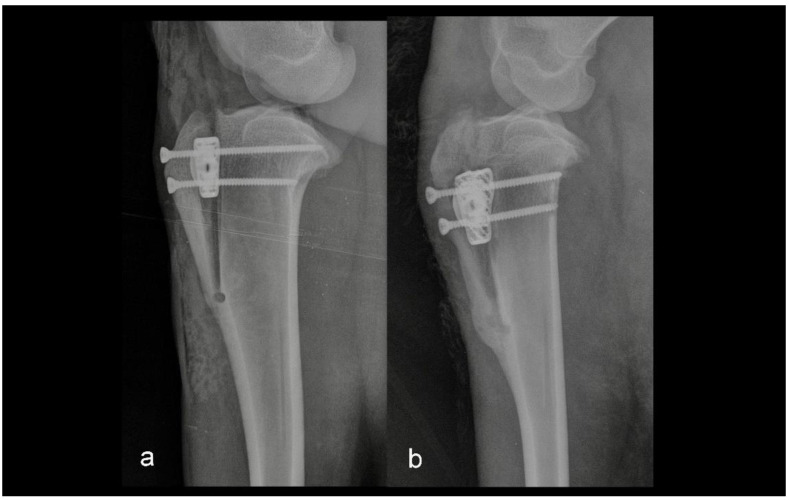
Mediolateral radiograph of the stifle in Group 2 immediately postoperatively (**a**) and at six months postoperatively (**b**). The osteotomy line was too close to the tibial tuberosity crest.

The second group consisted of five sheep in which the intraoperative or postoperative discrepancies from preoperative planning were found. Discrepancies were either the osteotomy line was too close to the tibial crest, or the tibial crest was cracked at the level of the Maquet hole (Figure 2 and Figure 3).

Our hypothesis was that the differences between the loads of failure in the two groups were significant.

## 2. Materials and Methods

### 2.1. Limb Preparation

Tibial bones (n = 10) were collected by disarticulation of the stifle and talocrural joints from adult Merino sheep weighing 45 kg (±5 kg) that were euthanized after a six-month follow-up period in an experimental in vivo TTA CF study [9]. All the animals underwent TTA CF surgery six months prior. Common tangent TTA measurements were used, and 6 mm width titanium TTA CF implants were used to assess the degree of advancement. Procedures in the study were conducted in accordance with the Local Ethical Committee for Experiments on Animals in Olsztyn (NR 07/2015 Olsztyn, Poland). The animals were obtained from the farm with registration nr PL 050649214001 (Barczewo, Poland). 

After imaging, the specimens were divided into two groups: **Group 1** = 5 tibiae where TTA CF surgeries were performed as assumed preoperatively; **Group 2** = 5 tibiae where cage misplacement or discrepancies from preoperative planning were found. In both groups the tibial tuberosity had been advanced with a 6 mm TTA CF titanium cage as described previously [9]. 

The skin and soft tissues were removed from the proximal aspect of the tibia to the talocrural joint. The tibiae were cut transversely in 1/3 of the distal part of the tibia with an oscillating saw. The specimens were individually wrapped in moistened towels that were soaked in isotonic saline and were delivered for testing within four hours. The samples were not frozen.

### 2.2. Mechanical Testing

**Figure 4:**
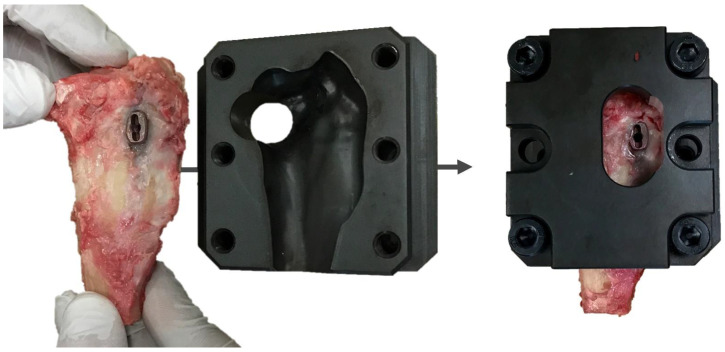
Image of a custom jig for stable fixation of the proximal tibia in the testing machine.

Before testing, the two 2.7 mm craniocaudal screws, which attached the cage to the tibia were unscrewed. The proximal tibiae were fixed into a custom jig, maintaining the medial surface of the tibiae and TTA CF cage in a horizontal position. The jig was made as a cast of the proximal tibia based on computed tomography, for stable fastening of the tibia to the test machine (Figure 4).

**Figure 5:**
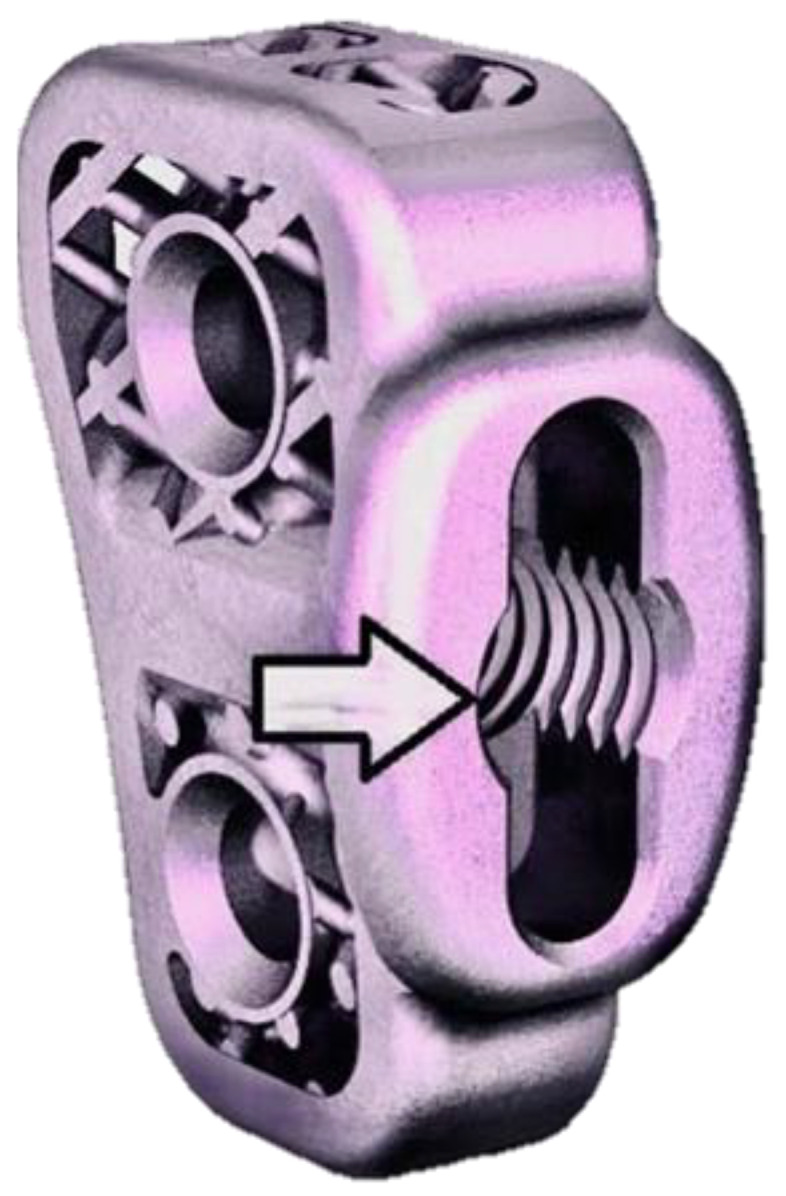
Image of the 6 mm TTA CF titanium implant. The end of the arrow points to a threaded hole on its medial longest edge for screwing the drill guide intraoperatively. The same threaded hole was used to fix the custom bar when performing biomechanical tests.

The TTA CF cage has a threaded hole on its medial longest edge for screwing the drill guide (Figure 5). 

**Figure 6:**
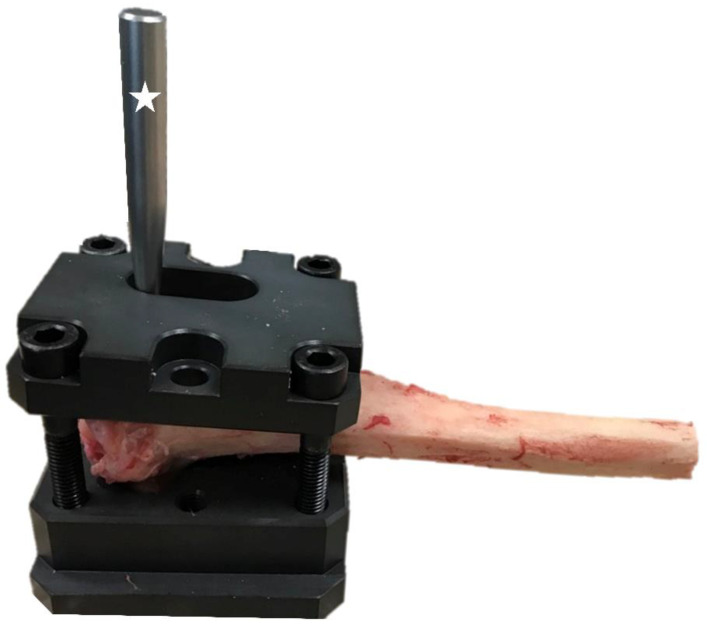
Specimen prepared for mechanical testing. A screwed custom bar is marked with an asterisk.

The same threaded hole served to fix the custom bar in it. A custom bar was screwed in a TTA CF threaded hole, perpendicular to the cage (Figure 6). 

**Figure 7:**
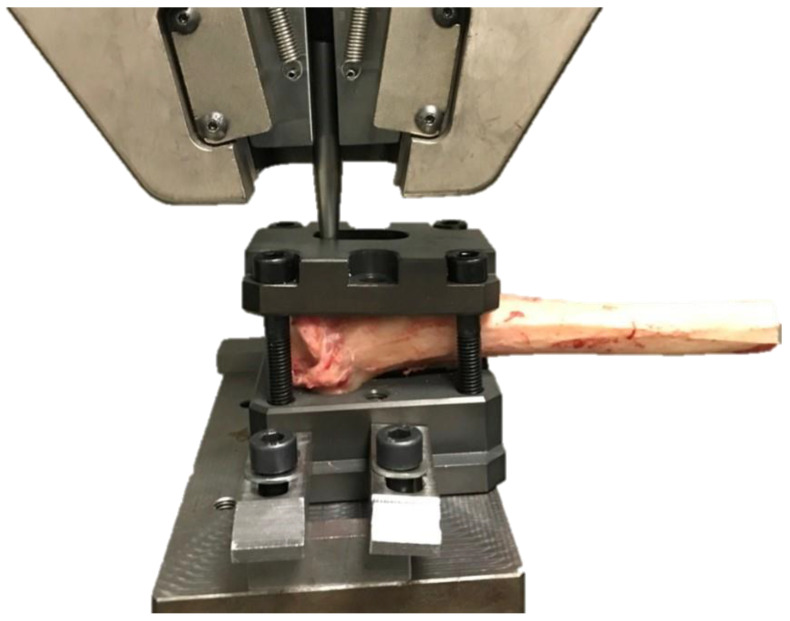
The tibia positioned in the testing machine. The jig with the proximal tibia was attached to the lower machine arm, and the bar was fixed in the upper frame of the electromechanical testing machine (MTS Insight 100 kN, Eden Prairie, MI, USA).

The custom jig and bar were fixed in the frame of the electromechanical testing machine (MTS Insight 100 kN, Eden Prairie, MI, USA) (Figure 7).

**Figure 8:**
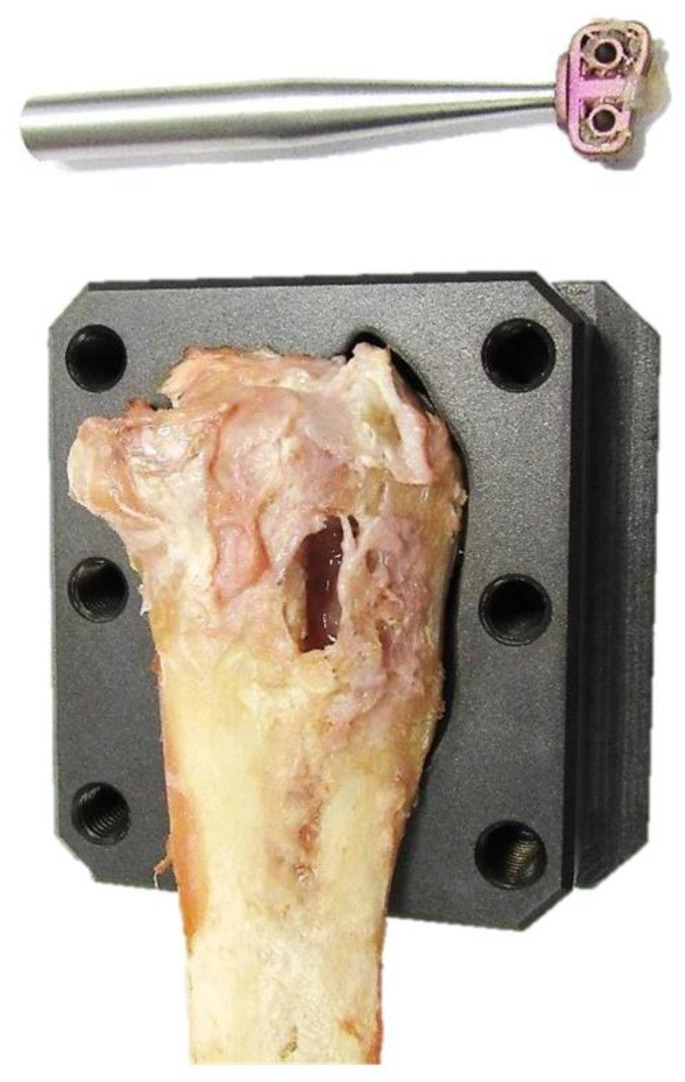
A torn TTA CF implant after biomechanical testing.

In both groups, tension was applied to the TTA CF cage at a rate of 5 mm/min, which was continuously applied until failure occurred (Figure 8). The applied force was directly perpendicular to the cage. The maximal loaded forces to pull out the cage and the displacement at maximal applied loads were recorded. 

### 2.3. Statistical Analysis

Statistical analysis was performed with SPSS software (IBM Corp. Released 2017. IBM SPSS Statistics for Windows, Version 26.0. Armonk, NY, USA: IBM Corp). The normality and homogeneity of the distribution were tested using the Shapiro–Wilk and Levene’s tests. The data did not follow the normal distribution. Therefore, the data are presented as the median and interquartile range (IQR) (Table 1). For study purposes, the work was calculated as work = 1/2 ∗ force [N] ∗ displacement [m]. The maximal loaded forces to pull out the cage, displacement of the implant, and the work that was required for rupture were compared between the two groups using the Mann–Whitney U test, and statistical significance was set at an alpha level of 0.05.

## 3. Results

A total of 10 ovine proximal tibiae were tested biomechanically by tearing out the implants from the bone. The mean maximal forces to pull out the cage was 878 ± 61 N and 330 ± 55 N in Group 1 and Group 2, respectively. The mean implant displacement under maximal load to failure was 2.6 mm and 2.2 mm in Groups 1 and 2, respectively. The detailed ultimate forces to pull out the cage and implant displacement under stress in both groups are reported in Table 2 and Table 3. 

We made a hypothesis that in specimen 1.4, 1.5, and 2.4 the outer part of the cortical bone was very hard, and, therefore, at first it gave a lot of resistance to the pulling force. At some point, the forces accumulated and displaced the cage with more power. Therefore, the displacement in mm per min was higher than in the other specimens.

**Figure 9:**
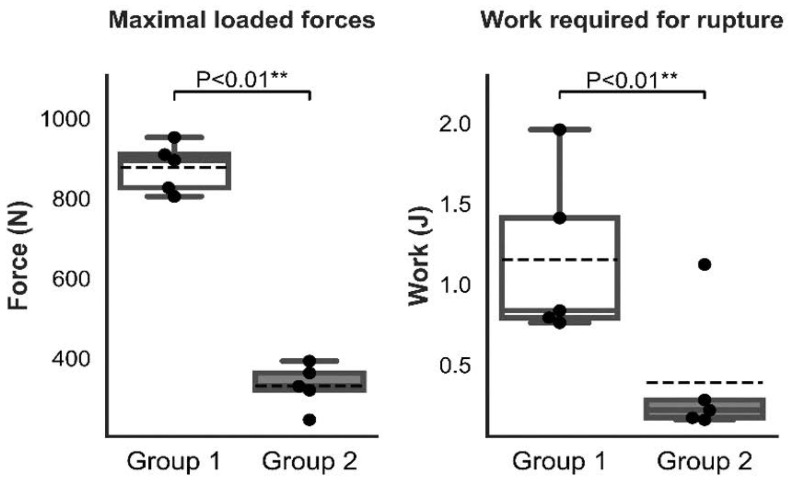
Graphic representation of the statistical analysis of the maximal loaded forces and work that was required for rupture in Groups 1 and 2. (“**” mean high significance, i.e., less than 0.01).

There was a significant difference between Group 1 and Group 2 in the maximal loaded forces to pull out the cage (Figure 9), however the difference in the displacement at the maximal loaded forces to pull out the cage was not significant between the groups. 

In Group 1 and Group 2, periosteal cracking failed at the level of osteotomy (Figure 8). For five tibiae in Group 2, the lower value of maximal loaded forces to pull out the implant was significantly related to the misplacement or discrepancy of the cage position from preoperative planning. None of the TTA CF implants in either group failed during the examination. 

## 4. Discussion

Rupture of CCL is the most common orthopedic disorder and results in the most common cause of hind limb lameness [1,2,3,21]. TTA and TPLO, both based on tibial osteotomies, are the most commonly used surgical procedures in dogs to achieve dynamic stability of the stifle joint during weight bearing [4,5,6,12,22]. The TTA technique is derived from the Maquet surgical technique and was first presented in 1976 to mitigate pain in chondromalacic and osteoarthritic patellofemoral joints in humans [13]. Conventional TTA and modifications are now well accepted surgical methods for CCL rupture repair [7,10,12,23]. 

TTA eliminates cranial femorotibial subluxation by ensuring a patellar tendon angle (PTA) of ≤90° when the stifle flexion angle is ±135° [5,6,22]. To achieve the desired PTA, the tibial tuberosity is osteotomized and cranially advanced by a measured width that is based on preoperative radiographs [5,6]. To maintain the assumed advancement, an appropriately sized TTA cage is placed in the osteotomy gap [5,6,8,9,10]. Foreseeable realization of the geometric targets of the TTA surgical technique is dependent on consistent and accurate preoperative planning [24]. Preoperative planning variables, such as the exact measurement of radiographic landmarks that are used for PTA determination, have been reported [17,23,25].

Original TTA is a traumatic surgery in which the tibial crest was completely osteotomized, and numerous implants and screws were used [26]. TTA modifications such as TTA Rapid [8], Modified Maquet Technique [19], Porous TTA [27], and TTA CF [8,9] use fewer implants to allow improved preservation of the blood supply that is needed for osteotomy gap healing. 

As there is a discrepancy between the desired tibial tuberosity advancement and the actually achieved advancement, the current tendency is to increase the TTA cage size that is specified by preoperative planning [28]. In our study with 10 cases, a 6 mm TTA CF cage was used. The TTA CF orthopedic technique was reported in an ovine model [9] and in 22 dogs that were affected with CCL rupture [10]. Sheep are becoming popular as biomechanical animal models in orthopedic research in veterinary and human medicine [29,30,31,32,33]. Sheep were used to research osteoarthrosis and osteoporosis treatment in humans and to assess the repair of fractures and ligament injuries [29]. The anatomy and biomechanics of the sheep stifle joint are similar to those of dogs [31,32,33]. 

Analyzing mechanical ex vivo experimental studies about modifications or new tibial tuberosity advancement techniques biomechanical effectiveness was evaluated by axial pressure on the tibial crest to test the strength and resistance of the fixation [16,18,19] or by pulling on the tuberosity [7,17,20] and checking what force will be needed to separate the tibial tuberosity. All of these biomechanical techniques use modified indirect pressure on the tibial tuberosity in order to verify the mechanical strength or a failure resistance. It is known from human literature that cortical bone porosity can vary from less than 5% to nearly 30%, which is mainly because of the volatility in the diameter, length, and number of Haversian and Volkmann canals, which may influence the bone biomechanical properties [34]. Our study is the first study to investigate the direct forces that are required to pull-out the TTA CF implant from the tibia. Our intention was to test the objective force that was needed to pull the implant out of the bone, not the indirect force acting on the tibial tuberosity.

In our biomechanical testing, the lower value of maximal loaded forces to pull extract the implant in Group 2 (330 ± 55 N) was significantly corresponded to the misplacement or discrepancy of the cage position from preoperative planning. That is twice as much force (613 ± 77 N) that was needed to detach the tibial tuberosity after classical forkless TTA with additional figure-of-eight cerclage wire [7] and four times less (1348 ± 504 N) than what was needed to detach the tuberosity by axial pressing of the tuberosity [19]. The higher forces (878 ± 61 N) that were needed to extract the TTA CF implant from the tibia occurred in Group 1 where the surgical procedures were performed as assumed preoperatively. These forces are comparable to the results for tearing the tuberosity after TTA by pulling on the patellar straight ligament [7,18,19].

Etchepareborde et al. (2010) calculated the load to failure after a 9 mm TTA procedure without plate fixation in three groups. In Group 1, where the distal part of the tibial crest was left attached and a figure of eight cerclage wires was added, the mean maximal tension load-to-failure was 1265 ± 275 N. In Group 2, where the distal part of the tibial crest was left attached but no additional fastening techniques were used, the mean maximal tension load-to-failure was 1123 ± 394 N. In Group 3, where the distal tibial crest was completely cut, the mean maximal tension load-to-failure was 613 ± 77 N [7]. The total detachment of the distal tibial crest results in significantly weaker resistance from a biomechanical point of view [7]. 

McCartney et al. (2019) used artificial bones for biomechanical experimental research on different variances of pins and tension band wiring as additional reinforcements during TTA surgery [18]. The highest resistance and strength using a single cycle to fail test with speeds of 50 mm/min and 1.5 N preload were found in the group with additional fixation of the TTA cage with both pins and tension bands. The mean strength in this group was 1.47 ± 0.07 N [18].

Lins et al. (2009) used ten pelvic limbs (n = 10) that were harvested by hip disarticulation for the mechanical resistance of TTA implants. Immediately after surgery, all the specimens were wrapped individually in 0.9% isotonic saline-soaked towels and frozen in plastic bags at −20 °C [16]. Etchepareborde et al. (2010), for modified TTA mechanical testing, collected 36 hind limbs (n = 36) by disarticulation of the coxofemoral joint, wrapped in moistened towels that were soaked in 0.9% saline, and stored in plastic bags at −20 °C [7]. Before the mechanical tests, the limbs were thawed to room temperature [7,16]. To test the new osteotomy for the modified Maquet technique, 156 pelvic limbs (n = 156) were collected immediately after euthanasia, and the dissected bone specimens were kept moist during the experiment by spraying with 0.9% isotonic saline solution [19]. The mean ultimate axial load to failure of the tibial tuberosity that was applied perpendicular to the tibial plateau was 1348 ± 504 N [19]. 

Ideally, for mechanical testing, samples should be tested immediately after euthanasia [7], but several studies have shown that freezing has no influence on the compression, bending, and torsion properties of the bone [35,36]. Nothing has been reported the influence on tension [7]. For this study, 10 ovine hind limbs (n = 11) were kept moist throughout the biomechanical tests but not frozen to reduce the effect of freezing on the biomechanical results.

All the TTA modifications restored the dynamic stability in CCL-injured joints by reducing the angle that was formed by the straight patellar ligament and tibial plateau and through tibial tuberosity osteotomy and advancing cranially. The value of advancement is based on preoperative measurements and the size of the tibia [15,17,19]. For this ex vivo research, a 6 mm width titanium TTA CF cage was used.

Correct preoperative planning and osteotomy line positioning are crucial to achieve satisfactory postoperative outcomes. The doubts regarding the safety of the tibial crest osteotomy that is prolonged distally in the tibial shaft or runs too cranially are not limited to the resistance of the tibial crest itself but to the resistance to shaft fracture of the tibia during weight bearing [19]. 

Our report has some limitations. The number of specimens could be higher. Future studies testing TTA modification surgical techniques that examined the strength that is needed to pull out an implant from the bone would be interesting. Another limitation is that specimens in our report were not compared against the control group that had a classical TTA. This presupposes that the data in this study are based on the interpretation of the presented TTA CF surgical technique that is not comparable with the control group or with other conventional TTA techniques. It would be interesting in further studies to use the same testing protocol to compare the maximal loaded forces that are needed to pull out the classical TTA cage from the tibia. Also, it would have been interesting to study the differences in the pull-out strength after the TTA procedure by pulling on the tuberosity in one group and by pulling forces directly on the TTA implant in a second group.

Additionally, in the present study we did not measure bone mineral density (BMD) around the TTA CF cage, which could influence the results. Our study does not examine the contribution of soft tissues to maintaining the implant in the tibia. 

## 5. Conclusions

The mean maximal loaded forces to pull out the cage was significantly lower in Group 2, where discrepancies from preoperative planning were found (878 ± 61 N vs. 330 ± 55 N). The lower forces that were needed to extract the TTA CF implant from the tibia can lead to the conclusion that biointegration of the implant is also weaker. Correct positioning of the osteotomy line and TTA CF implant is essential for good biointegrity and thus for limiting complications in the form of tibial tuberosity avulsion fracture or tibial shaft fracture.

## Figures and Tables

**Table 1:** Mean ± standard deviation.

	Group 1 n = 5	Group 2 n = 5
	Median (IQR)	Median (IQR)
Displacement [mm]	1.92 (1.64)	1.4 (0.56)
Maximal loaded forces [N]	**896 ^a^** (83)	**330 ^b^** (43)
Displacement [mm]	1.92 (1.64)	1.4 (0.56)
Work [J]	**0.84 ^a^** (0.02)	**0.22 ^b^** (0.11)

***(^a^ and ^b^ mean a significant difference between the groups at a significance level of at least 0.05).***

**Table 2:** Maximal forces and work to tear out the cage and implant displacement under stress in Group 1.

Specimen Number	Maximal Forces to Pull Out the Cage [N]	Displacement at Maximal Load-to-Failure [mm]	Work [J]
Specimen 1.1	827	1.92	0.79
Specimen 1.2	896	1.87	0.84
Specimen 1.3	953	1.60	0.76
Specimen 1.4	910	4.31	1.96
Specimen 1.5	805	3.51	1.41
Mean	878.2	2.64	1.15
Standard deviation	61.04	1.20	0.52

**Table 3:** Maximal forces and work to tear out the cage and implant displacement under stress in Group 2.

Specimen Number	Maximal Forces to Pull Out the Cage [N]	Displacement at Maximal Load-to-Failure [mm]	Work [J]
Specimen 2.1	363	1.21	0.22
Specimen 2.2	330	0.97	0.16005
Specimen 2.3	320	1.77	0.2832
Specimen 2.4	393	5.72	1.12398
Specimen 2.5	246	1.40	0.1722
Mean	330.4	2.21	0.39
Standard deviation	55.26	1.98	0.41

## Data Availability

Not applicable.

## Abbreviations

CCL—cranial cruciate ligament, TTA—tibial tuberosity advancement, TTA CF—tibial tuberosity advancement with cranial fixation, TPLO—tibial plateau leveling osteotomy, IBM—international business machines, SPSS—statistical package for the social sciences.
